# Automated Lung Sound Classification Using a Hybrid CNN-LSTM Network and Focal Loss Function

**DOI:** 10.3390/s22031232

**Published:** 2022-02-06

**Authors:** Georgios Petmezas, Grigorios-Aris Cheimariotis, Leandros Stefanopoulos, Bruno Rocha, Rui Pedro Paiva, Aggelos K. Katsaggelos, Nicos Maglaveras

**Affiliations:** 1 Laboratory of Computing, Medical Informatics and Biomedical—Imaging Technologies, Medical School, Aristotle University of Thessaloniki, GR 54124 Thessaloniki, Greece; petmezgs@auth.gr (G.P.); ncheimar@auth.gr (G.-A.C.); lstefano@auth.gr (L.S.); 2 Centre for Informatics and Systems, Department of Informatics Engineering, University of Coimbra, 3030-290 Coimbra, Portugal; bmrocha@dei.uc.pt (B.R.); ruipedro@dei.uc.pt (R.P.P.); 3 Department of Electrical and Computer Engineering, Northwestern University, Evanston, IL 60208, USA; a-katsaggelos@northwestern.edu

**Keywords:** lung sounds, crackles, wheezes, STFT, CNN, LSTM, focal loss, COPD, asthma

## Abstract

Respiratory diseases constitute one of the leading causes of death worldwide and directly affect the patient’s quality of life. Early diagnosis and patient monitoring, which conventionally include lung auscultation, are essential for the efficient management of respiratory diseases. Manual lung sound interpretation is a subjective and time-consuming process that requires high medical expertise. The capabilities that deep learning offers could be exploited in order that robust lung sound classification models can be designed. In this paper, we propose a novel hybrid neural model that implements the focal loss (FL) function to deal with training data imbalance. Features initially extracted from short-time Fourier transform (STFT) spectrograms via a convolutional neural network (CNN) are given as input to a long short-term memory (LSTM) network that memorizes the temporal dependencies between data and classifies four types of lung sounds, including normal, crackles, wheezes, and both crackles and wheezes. The model was trained and tested on the ICBHI 2017 Respiratory Sound Database and achieved state-of-the-art results using three different data splitting strategies—namely, sensitivity 47.37%, specificity 82.46%, score 64.92% and accuracy 73.69% for the official 60/40 split, sensitivity 52.78%, specificity 84.26%, score 68.52% and accuracy 76.39% using interpatient 10-fold cross validation, and sensitivity 60.29% and accuracy 74.57% using leave-one-out cross validation.

## 1. Introduction

Respiratory diseases constitute a major health issue and are responsible for a high rate of mortality worldwide [1]. In particular, chronic obstructive pulmonary disease (COPD) and lower respiratory system diseases are the number three and number four leading causes of death, respectively, accounting together for more than 5.8 million of deaths globally in 2019 [2]. In total, more than 1 billion people suffer from acute or chronic respiratory conditions that affect directly their quality of life, including COPD, asthma, acute lower respiratory tract infections, tuberculosis, pulmonary hypertension, sleep-disordered breathing and occupational lung diseases, among others.

Early diagnosis and patient monitoring are critical for the efficient management of respiratory diseases. In clinical practice, respiratory conditions are diagnosed through lung auscultation, which refers to listening to the patient’s lung sounds using a stethoscope. Lung sounds are conventionally divided into two categories—namely, normal and adventitious. Crackles, wheezes and squawks are the most common adventitious lung sounds that are heard over normal ones and their presence usually indicates a pulmonary disorder [3,4].

Nonetheless, the rapid growth of technology has enabled the massive increase in the amount of recorded data that sometimes makes traditional diagnosis infeasible, as it is time-consuming and requires high medical expertise. To address this problem, many studies have proposed various artificial intelligence (AI) techniques for the automated classification of lung sounds, including machine learning (ML) algorithms [5] such as support vector machines (SVM) and hidden Markov models (HMM), and deep learning (DL) architectures, such as convolutional neural networks (CNN), recursive neural networks (RNN), long short-term memory (LSTM) networks and residual networks (ResNet) [6].

Moreover, extensive research has been conducted concerning feature selection and extraction strategies for automated lung sound analysis and classification. Spectrograms, Mel-Frequency Cepstral Coefficients (MFCC), wavelet coefficients, chroma features and entropy-based features are some of the most commonly selected features when performing feature extraction from lung sounds.

In this paper we propose a hybrid DL architecture that combines CNN and LSTM architectures for the classification of normal and adventitious lung sounds. The rationale behind this approach is to use the CNN part of the network to extract deep features from the input data and reduce their dimensionality, and the LSTM part to identify and memorize the temporal dependencies in each input sequence. Additionally, the proposed model implements the focal loss (FL) function to achieve prediction error reduction and handle data imbalance. Training and evaluation of the model are performed on lung sounds originating from the ICBHI 2017 Respiratory Sound Database (RSD) [7], which includes normal sounds, as well as three types of adventitious lung sounds—namely, crackles, wheezes, and both crackles and wheezes.

The remainder of this paper is structured as follows: In Section 2, background knowledge for lung sound classification is presented and related state-of-the-art DL methods are reviewed. In Section 3, the proposed model is described in detail, while in Section 4, its performance is evaluated and discussed. Finally, in Section 5, conclusions of the study are summarized.

## 2. Related Work

During the last few years, considerable work has been done regarding automated lung sound classification. More specifically, Mazić et al. [8] applied feature sets containing MFCC, kurtosis and entropy features on a cascade SVM structure consisted of two parallel SVMs that use different values (5.0 and 2.0, respectively) for the gamma parameter of the radial basis function (RBF) kernel and fused their predictions to separate respiratory sounds into wheezes and non-wheezes, while Matsutake et al. [9] classified normal and abnormal respiratory sounds using HMMs and maximum likelihood estimation. A study by Sen et al. [10] compared the performances of a Gaussian mixture model (GMM) and an SVM classifier on recognizing normal and pathological lung sounds, while another study by Mendes et al. [11] proposed a logistic regression (LR) and a random forest (RF) classifier to evaluate the performance of 30 different features in detecting wheezes from respiratory signals.

Furthermore, Bokov et al. [12] employed an SVM classifier using spectral features to detect wheezes, while Chamberlain et al. [13] designed two SVM classifiers, one for wheezes identification and one for crackles identification using features that were extracted by a denoising autoencoder (DAE). A work by Sengupta et al. [14] implemented a multi-layer perceptron (MLP) and evaluated five different cepstral features to separate crackles and wheezes from normal lung sounds, while another work by Serbes et al. [15] implemented an SVM on short-time Fourier transform (STFT) and wavelet features to identify crackles and wheezes in lung sound recordings.

Also, Jakovljević et al. [16] fed MFCC features to an HMM for crackles and wheezes detection, while Bardou et al. [17] utilized MFCC and local binary pattern (LBP) features to train three different machine learning approaches—namely, an SVM, a k-nearest neighbor (KNN) model and a GMM, along with a CNN containing five convolutional layers, in order to classify respiratory sounds into seven different categories. An RNN has been introduced by Kochetov et al. [18] for the classification of lung sounds using MFCC features, while Chambres et al. [19] utilized MFCC features on an HMM and STFT features on an SVM for the same purpose.

Moreover, a study by Liu et al. [20] tested a log mel filterbank (LMFB) on a CNN for the identification of adventitious respiratory sounds, while another study by Minami et al. [21] combined STFT and continuous wavelet transform (CWT) features to train a CNN that predicts four classes of lung sounds. A bidirectional residual network (bi-ResNet) that uses STFT features and wavelet analysis was presented by Ma et al. [22] for lung sound classification, while Demir et al. [23] applied STFT features on a VGG16 and a SVM classifier for the same purpose.

In addition, Acharya and Basu [24] proposed a deep CNN-RNN model for the classification of respiratory sounds based on mel spectrograms, while Demir et al. [25] combined deep features extracted from a CNN with a linear discriminant analysis-random subspace ensembles (LDA-RSE) classifier to detect four different types of lung sounds. A work by Saraiva et al. [26] presented a CNN that processes MFCC features for crackles and wheezes detection, while another work by Gairola et al. [27] employed a CNN that utilized mel spectrograms to identify adventitious respiratory sounds.

At the same time, Ma et al. [28] introduced a non-local (NL) block into a ResNet and used STFT features for lung sound classification. Yang et al. [29] analyzed STFT features with a ResNet with squeeze and excitation (SE) and spatial attention (SA) blocks for the identification of abnormal lung sounds, while another study by Pham et al. [30] implemented a mixture-of-experts (MoE) block into a CNN structure and used mel spectrogram, gammatone-based spectrogram, MFCC and rectangular constant Q transform (CQT) features for the same purpose. Lastly, Nguyen and Pernkopf [31] implemented a ResNet to process mel spectrograms and classify respiratory sounds into four different categories.

## 3. Materials and Methods

### 3.1. Database

In the present study, respiratory sounds from the ICBHI 2017 Respiratory Sound Database were used. This is a highly cited database that includes a total of 5.5 h of recordings separated into 920 audio samples from 126 subjects. Each audio sample has been manually annotated by medical experts. In this way, there is a total of 6898 respiratory cycles, of which 3642 are normal, 1864 contain crackles, 886 contain wheezes and 506 contain both crackles and wheezes. The data collection was carried out by two research teams in two different countries, Portugal and Greece.

### 3.2. Preprocessing

**Figure 1:**
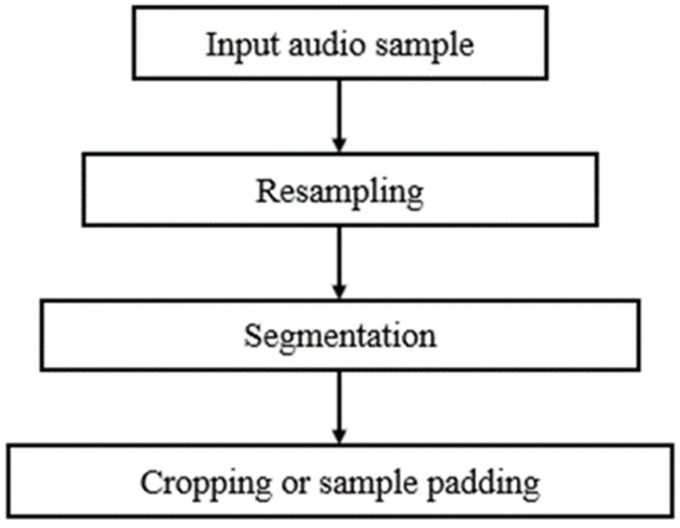
Block diagram of the lung sound preprocessing pipeline: at first audio samples are resampled at 4000 Hz, next each resampled signal is segmented into distinct respiratory cycles based on the experts’ annotations, and, finally, respiratory cycles of fixed duration are created using either signal cropping or sample padding.

The audio samples of the ICBHI 2017 Respiratory Sound Database were recorded using different sample rates between 4000 Hz and 44,100 Hz. As the signal of interest lies below 2000 Hz, it was safe to resample all recordings at 4000 Hz. Hereupon, the resampled recordings were segmented into distinct respiratory cycles based on the experts’ annotations. However, the duration of the respiratory cycles ranges from 0.2 s to 16 s with an average of 2.7 s. To address this issue, respiratory cycles of fixed duration have been extracted using a twofold strategy; cycles with a duration that exceeds 2.7 s were cropped preserving the first 2.7 s, while cycles with a duration lower than 2.7 s were expanded using sample padding [32]. Figure 1 presents the complete preprocessing pipeline that has been applied on the respiratory signals of the ICBHI 2017 Respiratory Sound Database.

### 3.3. Feature Extraction

**Figure 2:**
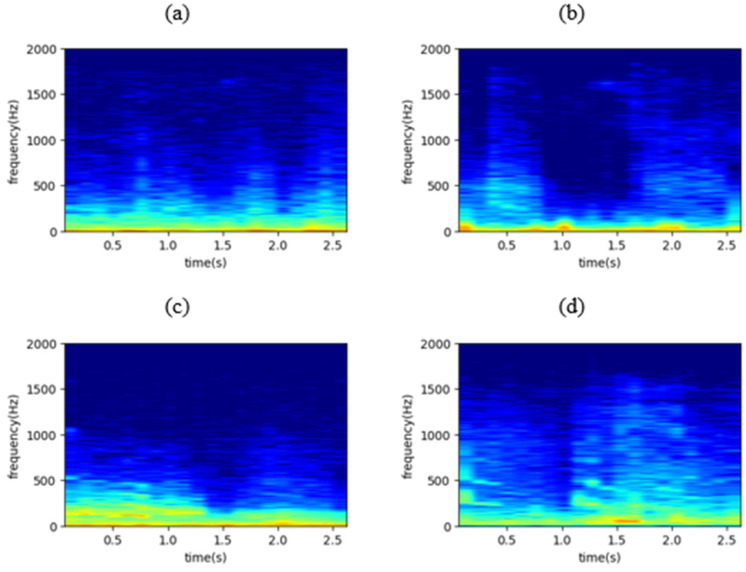
The spectrogram of (**a**) a normal respiratory cycle, (**b**) a respiratory cycle containing crackles, (**c**) a respiratory cycle containing wheezes, and (**d**) a respiratory cycle containing both crackles and wheezes, where x axis represents time in seconds and y axis represents frequency in Hz.

The short-time Fourier transform (STFT) [33] has been applied on the previously preprocessed respiratory cycles to identify their frequency content. The STFT is one of the most popular acoustic signal processing methods used for time-frequency analysis. Given a discrete signal *x*(*n*), its corresponding discrete-time STFT representation is calculated as:

(1) $$\mathrm{STFT}\{x(n)\}(m,\omega )\;\equiv \;X(m,\omega )=\sum_{n=-\infty }^{\infty }x\left(n\right)w\left(n-m\right)e^{-j\omega n}$$

where *w*(*n*) is the window function (usually Hanning or Gaussian). The STFT representation can reveal some useful information regarding the input waveform including its frequency components and their strength. In the present study, the squared magnitude of the windowed STFT representation, ${\left|X\left(m,\omega \right)\right|}^{2}$, also known as spectrogram, was implemented as input to the DL model. Figure 2 shows four representative spectrograms, one for each lung sounds class.

### 3.4. Convolutional Neural Networks

Convolutional neural networks (CNNs) [34] are one of the most popular DL architectures mainly used for classification tasks. They owe their name to the mathematical operation of convolution that is applied on the input data. The multidimensional discrete convolution is a linear operation that is given by:

(2) $$y(n_{1},n_{2})=(n_{1},n_{2})*w(n_{1},n_{2})=\sum_{k_{1}=-\infty }^{\infty }\sum_{k_{2}=-\infty }^{\infty }x\left(n_{1},n_{2}\right)w\left(n_{1}-k_{1},n_{2}-k_{2}\right)$$

where *x*(*n*_1_, *n*_2_) represents the input image, *w*(*n*_1_, *n*_2_) the impulse response of the filter and *y*(*n*_1_, *n*_2_) the output image. A typical CNN structure is composed of multiple hidden layers that are capable of adaptively learning the spatial hierarchy of data by extracting both high- and low-level patterns. The most common hidden layers are convolutional, pooling, batch normalization, dropout and fully-connected (or dense) layers.

The convolutional layers constitute the main structural elements of a CNN and extract features from the input data. In order to achieve that, they apply sets of filters, also known as kernels (the impulse response of the filter is the kernel), whose values are learned during training. These filters are two-dimensional (2D), since the one-dimensional (1D) lung sound time waveforms have been transformed into 2D images (spectrograms as in Figure 2) with the application of the STFT. The result of these convolutions of the input layer with the kernels generate feature (or activation) maps of higher abstraction as we move to deeper layers, identifying the most discriminative features for the task at hand (classification or regression).

On the other hand, the pooling layers are another part of a CNN and are usually placed right after each convolutional layer. They reduce the computational complexity of the network by conducting non-linear down-sampling on the extracted feature maps. Moreover, the batch normalization layers use batches to recenter and rescale the input data so that the training process is accelerated, while the dropout layers are responsible for preventing overfitting of the network by disabling some of its neurons. Finally, fully-connected layers are simple feed-forward neural networks (FNN) that are conventionally placed at the end of the network to map the aggregated activations of all previous layers into a class probability distribution by creating weighted connections between them.

### 3.5. Long Short-Term Memory Networks

Long short-term memory (LSTM) networks [35] are an improved version of recursive neural networks (RNN) [36] that memorize the temporal dynamics of sequential data for long time periods, a problem that classic RNNs cannot overcome easily. Regarding the structure of an LSTM network, it is comprised of memory blocks, also named cells, that are placed successively to handle the information flow. Each cell uses three gates, namely the forget, the input and the output gate, to control the process of adding or removing information from the network, and transfers two states to the next cell, the cell state and the hidden state.

### 3.6. Focal Loss Function

The loss function is a metric used while training a neural network to measure the error between the model’s predictions and the desired outputs. The rationale behind the use of the loss function is to minimize this error so that the model predicts correctly new inputs. The most known loss functions are mean squared error (MSE), that is typically applied when training regression models, and the cross-entropy (CE) loss that is widely used for classification.

In the present study, an improved version of the classic CE loss, called focal loss (*FL*) [37], is utilized. The *FL* function was recently proposed to solve the problem of class imbalance that is quite common in classification tasks. It is given by:

(3) $$FL=-\sum_{i=1}^{M}y_{i}{\left(1-{\hat{y}}_{i}\right)}^{\gamma }log({\hat{y}}_{i}),\;\gamma \geq 0$$

where $y_{i}\in \left\{1000,\;0100,\;0010,\;0001\right\}$ is the one-hot encoded ground-truth class label, ${\hat{y}}_{i}\in \left\{1000,\;0100,\;0010,\;0001\right\}$ the model’s estimated one-hot encoded probability for this class, *γ* the focusing parameter and ${\left(1-{\hat{y}}_{i}\right)}^{\gamma }$ the modulating factor. Due to the modulating factor ${\left(1-{\hat{y}}_{i}\right)}^{\gamma }$ the *FL* can automatically reduce the relative loss for easily classified examples (usually belonging to the bigger classes) during training and focus the model on harder, misclassified examples. The focusing parameter *γ* can adjust the rate at which the modulating factor ${\left(1-{\hat{y}}_{i}\right)}^{\gamma }$ affects the loss, thus higher values of *γ* increase the importance of correcting misclassified examples. Finally, *γ* = 2 has been found to give the best results, while for *γ* = 0 the *FL* is equivalent to the CE function.

### 3.7. The Proposed Model

In the present study, a hybrid 2D CNN-LSTM network incorporating the FL function to deal with data imbalance is proposed. The network receives as input respiratory cycles transformed into spectrogram images and classifies them into four distinct classes: normal, crackles, wheezes, and both crackles and wheezes. A similar approach but using a 1D CNN-LSTM architecture with completely different preprocessing filters was used in [38] for classification of 1D signal namely the ECG.

**Figure 3:**
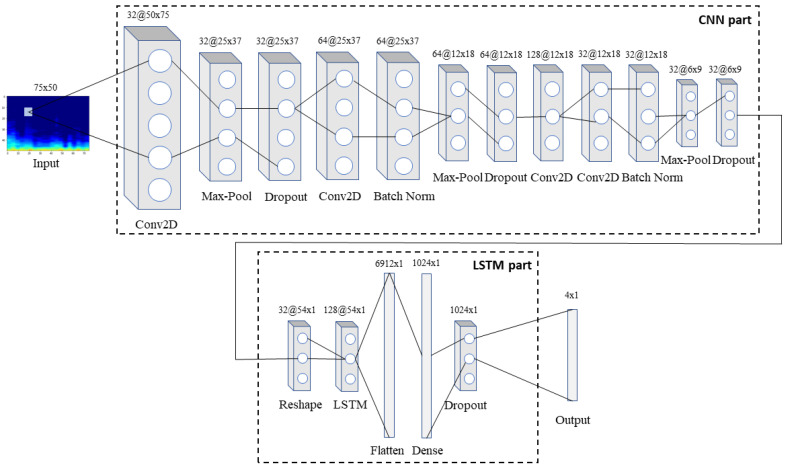
The architecture of the proposed model: the input spectrogram is passed through the CNN part of the network to extract features, which are then passed through the LSTM part so that the long-term dependencies between them are identified and memorized. Finally, the combined features are fed into the output layer that predicts the class to which the input belongs to.

Back to our model, this is comprised by an input layer, seventeen hidden layers and one output layer, that predicts classes, are placed in succession, as shown in Figure 3. More specifically, 1D respiratory signals are transformed into 2D RGB spectrogram images of size 75 × 50 pixels using STFT with a Hanning window and fed into the input layer. Firstly, the array is passed through the CNN part of the network containing in total four convolutional, three max-pooling, two batch normalization and three dropout layers. In this way, complex features are extracted from the input array, while at the same time, dimensionality reduction takes place as the input advances through the deeper layers of the network.

Next, the 3D (32 × 6 × 9) array that resulted from the above process and corresponds to 32 histograms extracted from the initial respiratory signal via the CNN part, each containing nine timesteps in the *x* axis, is reshaped into a 2D array (32 × 54 × 1) in order to be fed into the LSTM part of the network. This part is composed of one LSTM, one flattening, one fully-connected and one dropout layer, and is in charge of recognizing and memorizing the long-term dependencies in the data. Finally, an output layer that implements a softmax activation function predicts one of the four classes for each input spectrogram. Softmax is a generalization of the sigmoid function that is usually preferred as the output layer’s activation function in a multiclass classification network. Both the convolutional layers and the fully-connected layer utilize the non-linear rectified linear unit (ReLU) function as their activation function.

## 4. Results and Discussion

The design and implementation of the deep learning model for the automated classification of lung sounds were held in a Python 3.7.7 environment using the deep learning tool Keras. Tensorflow 2.1 was used as the backend of the Keras library. All experiments were performed on a desktop computer featuring an Intel Core i5-9600K 3.70 GHz CPU, a 16 GB RAM memory and an 8 GB NVIDIA GeForce RTX 2070 GPU.

**Figure 4:**
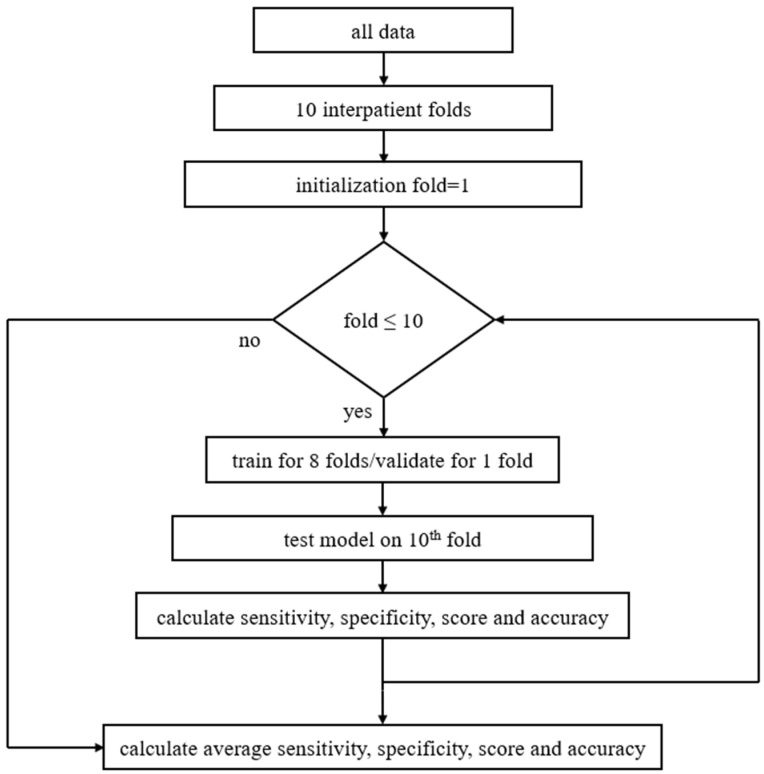
Block diagram of the proposed method including pseudocode: at first data are separated into ten interpatient folds, then 10-fold CV is being set, and the training process is repeated for all groups of folds. In each loop the model is trained and validated for nine folds, then tested on the 10th fold. Next, the micro-average values of the sensitivity, specificity, score, and accuracy are calculated. As soon as training is finished for all folds, average sensitivity, specificity, score, and accuracy are extracted.

The model was evaluated using three splitting strategies, namely the official ICBHI splitting [7], where 60% of data is used for training and 40% for testing, the interpatient 10-fold cross validation (CV), and the leave-one-out cross validation (LOOCV). Regarding the 10-fold CV, nine folds are used for algorithm development (eight for training and one for validation), while the last one is used for testing. In all three splitting methods different sets, i.e., training, validation, and testing, contain different patients, which makes the present study’s results more reliable. Figure 4 presents the proposed method regarding the model’s training and evaluation phase using 10-fold CV.

**Figure 5:**
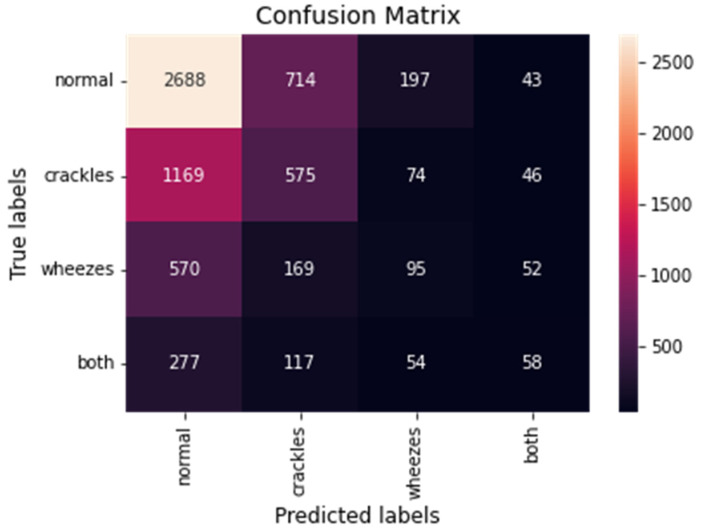
The overall confusion matrix for the CNN-LSTM model with *FL*.

Moreover, the model’s confusion matrix was calculated. The confusion matrix is an array where each row represents the actual class and each column the predicted class. The main diagonal of the matrix corresponds to the true positive predictions of the model. The overall confusion matrix of the proposed model is presented in Figure 5.

Nonetheless, the confusion matrix is not capable of quantifying the model’s performance; thus, the calculation of some well-known evaluation metrics, as follows, is required:

(4) $$\mathrm{sensitivity}=\frac{\mathrm{TP}\;}{\mathrm{TP}+\mathrm{FN}}$$

(5) $$\mathrm{specificity}=\frac{\mathrm{TN}\;}{\mathrm{TN}+\mathrm{FP}}$$

(6) $$\mathrm{score}=\frac{\mathrm{sensitivity}+\mathrm{specificity}\;}{2}$$

(7) $$\mathrm{accuracy}=\frac{\mathrm{TP}+\mathrm{TN}\;}{\mathrm{TP}+\mathrm{FP}+\mathrm{FN}+\mathrm{TN}}$$

where TP denotes true positive, TN true negative, FP false positive, and FN false negative predicted values. Due to the dataset’s class imbalance, for all metrics, the micro-average value was calculated. This means that the contributions of all classes are aggregated to compute the average metric.

Furthermore, multiple experiments were conducted in order to optimize the proposed model. In particular, the proposed model’s performance was evaluated while changing some of its parameters, namely the number of convolutional layers, the dropout rate, and the learning rate. All tests were performed using the interpatient 10-fold CV method to ensure the reliability of the results. In this way, Table 1 presents the overall sensitivity, specificity, score, and accuracy of the proposed model for different number of convolutional layers. The model was trained and tested using two to six convolutional layers in a structure similar to the one displayed in Figure 3. As can be observed, all evaluation metrics are maximized when four convolutional layers are utilized in the CNN part of the network (sensitivity 52.78%, specificity 84.26%, score 68.52% and accuracy 76.39%).

The model’s performance was evaluated for several dropout rates as well; that is, the frequency at which the dropout layers randomly set input units to zero at each step during training in order to prevent overfitting. Thus, as concluded from Table 2, all metrics reach their maximum values for a dropout rate of 0.2. Moreover, different learning rates were tested while training the model. As presented in Table 3, the best results are obtained for a learning rate of 0.0001.

In addition, the selection of the loss function seems to be crucial for the capability of the model to make accurate predictions on unseen data. Therefore, besides FL, the model was also evaluated using the classic CE loss function. The overall sensitivity specificity, score, and accuracy decreased, as expected, to 51.94%, 83.98%, 67.96%, and 75.97%, respectively.

Meanwhile, by using simpler architectures, the training time could be reduced, as the total network calculations become significantly fewer. Nonetheless, a separate test that was conducted using only the CNN part of the network resulted in evidently decreased overall sensitivity, specificity, score, and accuracy of 47.25%, 82.42%, 64.84% and 73.62%, respectively, which justifies why a hybrid CNN-LSTM network was preferred instead of implementing only a CNN.

Finally, a comparison between the performance of the proposed model and other methods used in recent relevant studies for the automated 4-class classification of lung sounds using the ICBHI 2017 Respiratory Sound Database is presented in Table 4. The proposed CNN-LSTM model with FL achieves state-of-the-art results using any of the three splitting strategies, the official 60/40 split, the interpatient 10-fold CV and the LOOCV. We strongly believe that the optimal combination of data preprocessing, DL model implementation (type and number of layers) and loss function selection (FL) is the main reason for this improvement in the results.

The accomplishments of the present study are the following: (i) the hybrid CNN-LSTM approach provides the best combination of performance (sensitivity, specificity, score, accuracy) in comparison with all previous relevant studies (including Pham et al. [30] as results using random 5-fold CV are not reliable), (ii) the proposed model performs well for a highly imbalanced dataset, (iii) the FL function delivers better results than the classic CE function for ECG classification, and (iv) the proposed method could be used for real-time lung sound classification as the prediction phase lasts only a few seconds.

On the other hand, the limitations of the present study could be summarized into two parts. Firstly, the proposed network was tested on classifying only three types of adventitious respiratory sounds (crackles, wheezes, and both crackles and wheezes); thus, it is incapable of detecting other lung sound categories, i.e., rhonchi and squawks. Secondly, although the ICBHI 2017 Respiratory Sound Database is well-established and one of the largest open-access lung sound databases, it contains only a limited number of respiratory cycles, so the results of the present study cannot be safely generalized. However, this is partially justified by the fact that the recording of respiratory sounds is a challenging process and compared to other physiological signals, i.e., ECG, fewer studies focus on them.

In conclusion, the present study was concentrated on the classification of lung sounds using data only from the ICBHI 2017 Respiratory Sound Database, while, at the same time, the feature extraction strategy was limited to spectrograms using the STFT transformation. In the future we intent to test the performance of the proposed method on new data from other databases and experiment with other features or possible combinations of them as well.

## 5. Conclusions

In this study, a hybrid CNN-LSTM model that implements FL was proposed in order to classify respiratory sounds into four categories using data from a highly-cited open-access database. More precisely, respiratory cycles were transformed into STFT spectrogram images and passed through a CNN that is capable of extracting their most significant features. Next, the extracted features were fed into an LSTM that identifies and memorizes the long-term dependencies between them, while FL was used to measure the model’s prediction error and deal with data imbalance.

To the best of our knowledge, this is the first study that combines the methodology of 2D CNN and LSTM networks with FL for the automated classification of respiratory sounds. The proposed model showed very promising results for three different data splitting strategies—namely, sensitivity 47.37%, specificity 82.46%, score 64.92%, and accuracy 73.69% for the official 60/40 split, sensitivity 52.78%, specificity 84.26%, score 68.52%, and accuracy 76.39% using interpatient 10-fold CV, and sensitivity 60.29% and accuracy 74.57% using LOOCV.

## Figures and Tables

**Table 1:** Proposed model’s performance for different number of convolutional layers.

No. of Convolutional Layers	Sensitivity	Specificity	Score	Accuracy
2	44.96%	81.65%	63.31%	72.48%
3	47.92%	82.64%	65.28%	73.96%
4	52.78%	84.26%	68.52%	76.39%
5	45.34%	81.78%	63.56%	72.67%
6	51.17%	83.72%	67.45%	75.58%

**Table 2:** Proposed model’s performance for different dropout rates.

Dropout Rate	Sensitivity	Specificity	Score	Accuracy
0	48.35%	82.78%	65.57%	74.17%
0.1	49.53%	83.18%	66.36%	74.76%
0.2	52.78%	84.26%	68.52%	76.39%
0.3	45.95%	81.98%	63.96%	72.98%
0.4	48.38%	82.79%	65.59%	74.19%
0.5	46.75%	82.25%	64.50%	73.37%

**Table 3:** Proposed model’s performance for different learning rates.

Learning Rate	Sensitivity	Specificity	Score	Accuracy
0.001	50.75%	83.58%	67.17%	75.38%
0.0001	52.78%	84.26%	68.52%	76.39%
0.00001	46.72%	82.24%	64.48%	73.36%

**Table 4:** Comparison between proposed model and relevant studies.

Study	Splitting Strategy	Method	Performance			
			Sen	Spe	Score	Acc
Serbes et al. [15]	official 60/40	SVM	-	-	-	49.86%
Jakovljević and Lončar-Turukalo [16]	official 60/40	HMM	-	-	39.56%	-
Kochetov et al. [18]	interpatient 5-fold CV	RNN	58.4%	73%	65.7%	-
Chambres et al. [19]	official 60/40	HMM	42.32%	56.69%	39.37%	49.50%
		SVM	48.90%	77.80%	49.86%	49.98%
Liu et al. [20]	random 75/25	CNN	-	-	-	81.62%
Minami et al. [21]	official 60/40	CNN	28%	81%	54%	-
Ma et al. [22]	official 60/40	bi-ResNet	31.12%	69.20%	50.16%	52.79%
	random 10-fold CV		58.54%	80.06%	69.30%	67.44%
Acharya and Basu [24]	interpatient 80/20	CNN-RNN	48.63%	84.14%	66.38%	-
Demir et al. [25]	10-fold CV	VGG16	-	-	-	63.09%
		SVM	-	-	-	65.5%
Saraiva et al. [26]	random 70/30	CNN	-	-	-	74.3%
Gairola et al. [27]	official 60/40	CNN	40.1%	72.3%	56.2%	-
	interpatient 80/20		53.7%	83.3%	68.5%	-
Ma et al. [28]	official 60/40	ResNet + NL	41.32%	63.20%	52.26%	-
	interpatient 5-fold CV		63.69%	64.73%	64.21%	-
Yang et al. [29]	official 60/40	ResNet + SE + SA	17.84%	81.25%	49.55%	-
Pham et al. [30]	official 60/40	CNN-MoE	26%	68%	47%	-
	random 5-fold CV		68%	90%	79%	-
Nguyen and Pernkopf [31]	official 60/40	ResNet	37.24%	79.34%	58.29%	-
This work	official 60/40	CNN-LSTM with FL	47.37%	82.46%	64.92%	73.69%
	Interpatient 10-fold CV		52.78%	84.26%	68.52%	76.39%
	LOOCV		60.29%	-	-	74.57%

## Data Availability

Not applicable.
